# mbend: an R package for bending non-positive-definite symmetric matrices to positive-definite

**DOI:** 10.1186/s12863-020-00881-z

**Published:** 2020-09-03

**Authors:** Mohammad Ali Nilforooshan

**Affiliations:** Livestock Improvement Corporation, Private Bag 3016, Hamilton, 3240 New Zealand

**Keywords:** Matrix, Positive-definite, Bending, Eigenvalue, R

## Abstract

**Background:**

R package mbend was developed for bending symmetric non-positive-definite matrices to positive-definite (PD). Bending is a procedure of transforming non-PD matrices to PD. The covariance matrices used in multi-trait best linear unbiased prediction (BLUP) should be PD. Two bending methods are implemented in mbend. The first is an unweighted bending with small positive values in a descending order replacing negative eigenvalues (LRS14), and the second method is a weighted (precision-based) bending with a custom small positive value (ϵ) replacing smaller eigenvalues (HJ03). Weighted bending is beneficial, as it relaxes low precision elements to change and it reduces or prohibits the change in high precision elements. Therefore, a weighted version of LRS14 was developed in mbend. In cases where the precision of matrix elements is unknown, the package provides an unweighted version of HJ03. Another unweighted bending method (DB88) was tested, by which all eigenvalues are changed (eigenvalues less than ϵ replaced with 100 × ϵ), and it is originally designed for correlation matrices.

**Results:**

Different bending procedures were conducted on a 5 × 5 covariance matrix (**V**), **V** converted to a correlation matrix (**C**) and an ill-conditioned 1000 × 1000 genomic relationship matrix (**G**). Considering weighted distance statistics between matrix elements before and after bending, weighting considerably improved the bending quality. For weighted and unweighted bending of **V** and **C**, HJ03–4 (HJ03, ϵ = 10^−4^) performed the best. HJ03–2 (HJ03, ϵ = 10^−2^) ranked better than LRS14 for **V**, but not for **C**. Though the differences were marginal, LRS14 performed the best for **G**. DB88–4 (DB88, ϵ = 10^−4^) was used for unweighted bending and it ranked the last. This method could perform considerably better with a lower ϵ.

**Conclusions:**

R package mbend provides necessary tools for transforming symmetric non-PD matrices to PD, using different methods and parameters. There were benefits in both weighted bending and small positive values in a descending order replacing negative eigenvalues. Thus, weighted LRS14 was implemented in mbend. Different bending methods might be preferable for different matrices, depending on the matrix type (covariance vs. correlation), number and the magnitude of negative eigenvalues, and the matrix size.

## Background

Even in their simplest form, multivariate animal models rely on genetic and residual variance-covariance matrices across traits [1]. These matrices are in the order of the number of traits in the model, and their inverses are incorporated in the mixed model equations. Inversion of a matrix is often done using Cholesky decomposition, which requires the matrix to be positive-definite (PD). For models including additional random effects (e.g., animal permanent environment, maternal genetic, and maternal permanent environment), additional covariance matrices and their inverses are also required. To date, restricted (or residual) maximum likelihood (REML) [2] is the preferred method for estimating the variance components associated with the model. REML estimates are always PD, but the starting matrices need to be PD. Elements of these matrices are usually from different sources of information and the resulting matrices are, therefore, likely to be non-PD [3].

Another complexity is the estimation of covariance matrices for several traits at a time. This complexity increases by both the number of traits, and the number of random effects. In many situations, there might not be enough data points to support accurate inferences about all the variance components, simultaneously. Therefore, when many traits are included, variance components are usually estimated for subsets of traits [4]. The assembly of these small matrices to a large matrix, together with best guesses for missing elements (from literature or phenotypic covariances and heritabilities) can be non-PD.

The procedure of “bending”, which involves modifying eigenvalues of a non-PD matrix, was first introduced by Hayes and Hill [5]. Latter studies in the field of animal breeding and genetics presented different bending procedures. Jorjani et al. [4] introduced weighted bending, where a weight matrix is provided for the matrix to be bent. Meyer and Kirkpatrick [6] developed a bending procedure, based on penalized maximum likelihood and reducing bias in the estimates of canonical heritabilities (the eigenvalues of **P**^−1^**G**, where **P** and **G** are the phenotypic and genetic covariance matrices, respectively). Also, Schaeffer [3] introduced a bending procedure, which involves changing negative eigenvalues to small positive values and reconstructing the matrix. A bending method for correlation matrices (also called smoothing), used in the field of psychology, involves changing all eigenvalues [7]. All these bending methods aim to produce a PD matrix, least deviated from the original non-PD matrix.

The aim of this study was to introduce R package mbend [8], which is a free and open source tool for bending symmetric non-PD matrices to PD, based on eigenvalue modification of the non-PD matrix. Comparison of different methods were provided, using example covariance and correlation matrices, as well as an artificial ill-conditioned genomic relationship matrix (**G**). R package mbend covers methods of Jorjani et al. [4] and Schaeffer [3], as well as extensions to these methods.

## Implementation

R package mbend [8] is written in R, and it is available on CRAN repository (https://cran.r-project.org) and can be installed, using the command install.packages(“mbend”). In this study, the functionality of this package was presented using the same 5 × 5 non-PD matrix used by Jorjani et al. [4] and Schaeffer [3].

$$ \mathbf{V}=\left[\begin{array}{ccccc}100& 95& 80& 40& 40\\ {}& 100& 95& 80& 40\\ {}& & 100& 95& 80\\ {}& & & 100& 95\\ {} Sym.& & & & 100\end{array}\right] $$

To study bending on a correlation matrix, **C** = **V**/100 was used. Jorjani et al. [4] used a matrix of weighting factors (**W**) as the reciprocal of the number of animals involved in the estimation of variance components. The same matrix is also used in this study for weighted bending.

$$ \mathbf{W}=1/\left[\begin{array}{ccccc}1000& 500& 20& 50& 200\\ {}& 1000& 500& 5& 50\\ {}& & 1000& 20& 20\\ {}& & & 1000& 200\\ {} Sym.& & & & 1000\end{array}\right] $$

For further comparisons, an ill-conditioned **G** matrix was constructed using random genotypes on 5000 SNP and 1000 animals, without any quality control checks, and 10 duplicated genotypes, which resulted in a **G** with 5 negative eigenvalues (ranging between –224e–17 to − 3.5e–17) and 357 eigenvalues between 0 and 1. Matrix **G** was constructed using method 1 of VanRaden [9]. The code for constructing the **G** matrix together with all the (R) code used in this study are provided in the data repository.

R package mbend [8] was used throughout this study. It provides different methods for weighted and unweighted bending of symmetric non-PD matrices to PD. Two bending methods of Jorjani et al. [4] and Schaeffer [3] and extensions to them are implemented in R package mbend [8].

### Method of Jorjani et al. [4]

This method (HJ03) is an iterative weighting procedure that converts a non-PD covariance matrix to a PD matrix at convergence. It considers different precision associated with different elements of the covariance matrix. Therefore, minimising the change in matrix elements with high accuracy, through a weight matrix. Given the non-PD covariance matrix **V**, the weight matrix **W**, and a small positive real number (e.g., ϵ = 10^−4^), the procedure is as follows:

1. Decompose **V**_*n*_ to **U**_*n*_**D**_*n*_**U**_*n*_′, where **U**_*n*_ and **D**_*n*_ are the matrix of eigenvectors and diagonal matrix of eigenvalues, and *n* is the iteration number.
2. Replace eigenvalues less than ϵ with ϵ in **D**_*n*_ to get **Δ**_*n*_.
3. **V**_*n* + 1_ = **V**_*n*_ − [**V**_*n*_ − **U**_*n*_**Δ**_*n*_**U**_*n*_′] ∘ **W**, where ∘ is the Hadamard function.
4. Repeat until **V**_*n* + 1_ is PD.

The smaller the *w*_*ij*_ (element in **W**), the higher the relative certainty about *v*_*ij*_ (element in **V**). Accordingly, to retain an element of the matrix fixed during bending, that element would receive a weight of zero. In comparison with the weighted procedure, in the unweighted procedure, **V**_*n* + 1_ = **U**_*n*_**Δ**_*n*_**U**_*n*_′. If no weight matrix is provided, the program performs an unweighted bending. Also, if the matrix is already PD, the program returns a message that “No action was required. The matrix is positive-definite”.

Jorjani et al. [4] extended their weighted bending method for covariance matrices to correlation matrices. R package mbend took a different approach for correlation matrices. First, it automatically recognises correlation matrices by checking all diagonal values against 1. Second, it treats correlation matrices as covariance matrices that should keep their diagonal elements constant during bending, by setting *w*_*ii*_ = 0. To avoid this restriction for a non-correlation matrix with all diagonal elements of 1 (e.g., a phenotypic matrix with variables standardised for phenotypic variances of 1), simply the matrix is multiplied by a positive constant, and then the resulting bent matrix is divided by that constant. A different approach that could be used for correlation matrices was treating them as covariance matrices and transforming the bent matrix ($\hat{\mathbf{V}}$) to $\mathbf{T}\hat{\mathbf{V}}\mathbf{T}^{\prime }$, where ${\mathbf{T}}^2=\mathit{\operatorname{diag}}\left(1/\mathit{\operatorname{diag}}\left(\hat{\mathbf{V}}\right)\right)$.

### Method of Schaeffer [3]

This method (LRS14) is an unweighted bending procedure, which means that different matrix elements are of the same precision. Negative eigenvalues are replaced with small positive values that are in a descending order (unlike equal values for HJ03). In this method, each of the *m* negative eigenvalues (*λ*_*i*_) is replaced with *ρ*(*s* − *λ*_*i*_)^2^/(100*s*^2^ + 1), where *ρ* is the smallest positive eigenvalue and $s=2\sum \limits_{i=1}^m{\lambda}_i$. In R package mbend, a weighted bending derivate of LRS14 is implemented by combining this method with HJ03 [8].

### Method of Bock et al. [7]

This method (DB88) is used for bending correlation matrices [7]. In this method, eigenvalues smaller than ϵ are replaced with 100 × ϵ. Also, unlike Jorjani et al. [4] and Schaeffer [3], eigenvalues greater than ϵ are changed to sum the number of those eigenvalues. The logic behind it might be that the sum of eigenvalues in a PD correlation matrix is equal to the size (trace) of the matrix. This method is implemented in function cor.smooth of R package psych (psych::cor.smooth) [10]. As this method is designed for bending correlation matrices, covariance matrices are first transformed to correlation matrices, after bending, the resulting matrix is transformed back to a covariance matrix (i.e., $\mathbf{T}\hat{\mathbf{C}}\mathbf{T}^{\prime }$, where **T**^2^ =  *diag* (*diag*(**V**))). That means, only the off-diagonal elements of the matrix change by bending.

### Deviation and correlation

R package mbend returns the following statistics:

1. Minimum deviation ($\hat{\mathbf{V}}-\mathbf{V}$), together with matrix indices of the element
2. Maximum deviation, together with matrix indices of the element
3. Average deviation of the upper triangle elements
4. Average absolute deviation of the upper triangle elements (AAD)
5. Weighted AAD
6. Correlation between the upper triangle elements
7. Weighted correlation between the upper triangle elements
8. Root of mean squared deviation of the upper triangle elements (RMSD)
9. Weighted RMSD
  $$ {\displaystyle \begin{array}{rr}\mathrm{AAD}& =\frac{\sum_{i=1}^n{\sum}_{j=1,j\ge i}^n\left|{\hat{v}}_{ij}-{v}_{ij}\right|}{n\left(n+1\right)/2},\\ {}\mathrm{Weighted}\ {\mathrm{AAD}}_{\left({w}_{ij}>0\right)}& =\frac{\sum_{i=1}^n{\sum}_{j=1,j\ge i}^n\left|\left({\hat{v}}_{ij}-{v}_{ij}\right)/{w}_{ij}\right|}{\sum_{i=1}^n{\sum}_{j=1,j\ge i}^n\left(1/{w}_{ij}\right)},\\ {}\mathrm{RMSD}& =\sqrt{\frac{\sum_{i=1}^n{\sum}_{j=1,j\ge i}^n{\left({\hat{v}}_{ij}-{v}_{ij}\right)}^2}{n\left(n+1\right)/2}},\\ {}{\mathrm{Weighted}\ \mathrm{RMSD}}_{\left({w}_{ij}>0\right)}& =\sqrt{\frac{\sum_{i=1}^n{\sum}_{j=1,j\ge i}^n{\left(\left({\hat{v}}_{ij}-{v}_{ij}\right)/{w}_{ij}\right)}^2}{\sum_{i=1}^n{\sum}_{j=1,j\ge i}^n\left(1/{w}_{ij}^2\right)}},\end{array}} $$

where, *n* is the size of the matrix, and *w*_*ij*_, *v*_*ij*_ and ${\hat{v}}_{ij}$ are the elements of **W**, **V** and $\hat{\mathbf{V}}$, respectively. The weighted statistics (weighted correlation, weighted AAD and weighted RMSD) are calculated for matrix elements with *w*_*ij*_ > 0. For correlation matrices, upper triangle elements did not include diagonal elements. Thus,

$$ {\displaystyle \begin{array}{rr}\mathrm{AAD}& =\frac{\sum_{i=1}^{n-1}{\sum}_{j=2,j\ge i}^n\left|{\hat{v}}_{ij}-{v}_{i\mathrm{j}}\right|}{n\left(n-1\right)/2},\\ {}\mathrm{Weighted}\ {\mathrm{AAD}}_{\left({w}_{ij}>0\right)}& =\frac{\sum_{i=1}^{n-1}{\sum}_{j=2,j\ge i}^n\left|\left({\hat{v}}_{ij}-{v}_{ij}\right)/{w}_{ij}\right|}{\sum_{i=1}^{n-1}{\sum}_{j=2,j\ge i}^n\left(1/{w}_{ij}\right)},\\ {}\mathrm{RMSD}& =\sqrt{\frac{\sum_{i=1}^{n-1}{\sum}_{j=2,j\ge i}^n{\left({\hat{v}}_{ij}-{v}_{ij}\right)}^2}{n\left(n-1\right)/2}},\\ {}{\mathrm{Weighted}\ \mathrm{RMSD}}_{\left({w}_{ij}>0\right)}& =\sqrt{\frac{\sum_{i=1}^{n-1}{\sum}_{j=2,j\ge i}^n{\left(\left({\hat{v}}_{ij}-{v}_{ij}\right)/{w}_{ij}\right)}^2}{\sum_{i=1}^{n-1}{\sum}_{j=2,j\ge i}^n\left(1/{w}_{ij}^2\right)}}.\end{array}} $$

### Function “bend”

R package mbend has a function called bend that is used with the syntax: bend (inmat, wtmat, reciprocal = FALSE, max.iter = 10,000, small.positive = 0.0001, method = “hj”). If any of the last 4 arguments are missing, the function will use the default value (FALSE, 10000, 0.0001, and “hj”, respectively). Arguments inmat and wtmat are for the matrix to be bent and the weight matrix. If wtmat is missing, an unweighted bending is performed. Argument reciprocal takes TRUE or FALSE as input, and if TRUE, reciprocals of **W** elements are used. Where *w*_*ij*_ = 0, it would remain 0. This argument is ignored if no weight matrix is provided to the function. The maximum number of iterations is defined by max.iter. The argument small.positive is used for HJ03 and ignored for LRS14. It is a user-defined small positive value (ϵ) replacing smaller eigenvalues in **D** [4]. Argument method takes “hj” or “lrs” for HJ03 and LRS14, respectively.

The function returns $\hat{\mathbf{V}}$, eigenvalues of **V** and $\hat{\mathbf{V}}$, and the statistics described in “Deviation and correlation”, all in a single list. Where weighted bending is applied, the number of upper triangle elements with *w*_*ij*_ > 0 (w_gt_0) is reported, as the weighted statistics rely on these observations. An example of weighted bending a correlation matrix, using bend function of mbend package is provided in the Additional file 1.

## Results

### The covariance matrix (V)

Matrix **V** is a non-PD covariance matrix. The following command in R shows eigenvalues from the eigendecomposition of **V**.

> round (eigen(V)$values, 2)

[1] 399.48 98.52 23.65 -3.12 -18.52.

Table 1 shows deviations and correlations between **V** and $\hat{\mathbf{V}}$ for unweighted bending, using HJ03–2 (HJ03, ϵ = 10^−2^), HJ03–4 (HJ03, ϵ = 10^−4^), LRS14 and DB88–4 (DB88, ϵ = 10^−4^). The unweighted HJ03 is like the iterative spectral method [11], which is a simplified form of altering projections method [12]. The difference between the unweighted HJ03 and the iterative spectral method is that, in the iterative spectral method, negative eigenvalues are replaced with the small positive value, but in HJ03, eigenvalues smaller than the small positive value are replaced with the small positive value. An unweighted HJ03 is equivalent to HJ03 with **W** = **11**′. Though some differences were small, the methods can be ranked from HJ03–4 being the best performer to HJ03–2, LRS14 and DB88–4. All the processes converged in 1 iteration.

**Table 1:** Deviation and correlation between the upper triangle elements of *V*_(5 × 5)_ (the covariance matrix) and its unweighted bent matrix

Statistics	HJ03–2^*a*^	HJ03–4^*b*^	LRS14^*c*^	DB88–4^*d*^
Min (dev.)	−5.9320	−5.9296	−5.9370	−10.9546
Max (dev.)	6.5016	6.4973	6.5418	2.6427
Mean (dev.)	0.7241	0.7235	0.7330	−2.9890
AAD	3.3754	3.3727	3.4087	3.6937
Correlation	0.9856	0.9856	0.9855	0.9833
RMSD	3.9300	3.9275	3.9542	5.0784
Number of iterations	1	1	1	1

dev. = bend(**V**) – **V**; AAD = average absolute deviation; RMSD = root of mean squared deviation; ^*a*^ Method of Jorjani et al. [4] with *ϵ* = 10^−2^; ^*b*^ Method of Jorjani et al. [4] with *ϵ* = 10^−4^; ^*c*^ Method of Schaeffer [3]; ^*d*^ Method of Bock et al. [7] with *ϵ* = 10^−4^

Table 2 shows deviations and correlations between **V** and $\hat{\mathbf{V}}$ for weighted bending, using HJ03–2, HJ03–4 and LRS14. The weighted bending procedures produced the same weighted correlation coefficients (0.9955) between the upper triangle elements of **V** and $\hat{\mathbf{V}}$. As expected, HJ03–4 performed better than HJ03–2. LRS14 produced the closest mean of deviation to zero. However, it performed worse than HJ03–2 (considering the range of deviations, weighted AAD, weighted RMSD, and the number of iterations to convergence). LRS14 took the maximum (787) number of iterations to converge. However, it was not a concern, as the convergence was made in less than 0.2 s, due to the small size of **V**.

**Table 2:** Deviation and correlation between the upper triangle elements of *V*_(5 × 5)_ (the covariance matrix) and its weighted (using *W*_(5 × 5)_) bent matrix

Statistics	HJ03–2^*a*^	HJ03–4^*b*^	LRS14^*c*^
Min (dev.)	−20.0068	−20.0008	−19.9923
Max (dev.)	5.8467	5.8456	5.8638
Mean (dev.)	−1.7167	−1.7161	−1.7158
AAD	3.6262	3.6253	3.6338
Weighted AAD	0.6102	0.6100	0.6122
Correlation	0.9623	0.9623	0.9622
Weighted correlation	0.9955	0.9955	0.9955
RMSD	6.3706	6.3687	6.3748
Weighted RMSD	0.5328	0.5327	0.5347
Number of iterations	248	428	787

dev. = bend(**V**) – **V**; AAD = average absolute deviation; RMSD = root of mean squared deviation; ^*a*^ Method of Jorjani et al. [4] with ϵ = 10^−2^; ^*b*^ Method of Jorjani et al. [4] with ϵ = 10^−4^; ^*c*^ Method of Schaeffer [3]

The program provides weighted statistics for weighted bending. Those statistics were manually calculated for unweighted bending of **V** and **C** matrices. The code is available in the data repository, and the results are provided in Table S1. Comparing Tables 1, 2 and S1 shows lower weighted AAD and weighted RMSD, and higher weighted correlation for weighted bending compared to unweighted bending, at the cost of greater deviations, AAD and RMSD, and lower correlation coefficients.

### The correlation matrix (C)

Table 3 shows deviations and correlations between **C** and $\hat{\mathbf{C}}$ for unweighted bending, for different methods. The differences between results from different methods were marginal. Overall, the methods can be ranked from HJ03–4 being the best performer to LRS14, HJ03–2 and DB88–4. The criteria for this ranking were the mean of deviations, AAD and RMSD. Likely, DB88 with ϵ < 10^−4^ could perform as good as HJ03–4 and LRS14, because in this method, eigenvalues smaller than ϵ are replaced with 100 × ϵ.

**Table 3:** Deviation and correlation between the upper triangle (excluding diagonal) elements of *C*_(5 × 5)_ (the correlation matrix) and its unweighted bent matrix

Statistics	HJ03–2^*a*^	HJ03–4^*b*^	LRS14^*c*^	DB88–4^*d*^
Min (dev.)	−0.0826	−0.0797	−0.0788	−0.1095
Max (dev.)	0.0725	0.0702	0.0717	0.0264
Mean (dev.)	−0.0200	−0.0194	−0.0204	−0.0448
AAD	0.0490	0.0475	0.0491	0.0554
Correlation	0.9841	0.9853	0.9854	0.9896
RMSD	0.0537	0.0520	0.0530	0.0622
Number of iterations	4	13	39	1

dev. = bend(**V**) – **V**; AAD = average absolute deviation; RMSD = root of mean squared deviation; ^*a*^ Method of Jorjani et al. [4] with ϵ = 10^−2^; *b* Method of Jorjani et al. [4] with ϵ = 10^−4^; ^*c*^ Method of Schaeffer [3]; ^*d*^ Method of Bock et al. [7] with ϵ = 10^−4^

Table 4 shows deviations and correlations between **C** and $\hat{\mathbf{C}}$ for weighted bending, using HJ03–2, HJ03–4 and LRS14. HJ03–4 and LRS14 performed almost equally better than HJ03–2. It took LRS14 a considerably greater number of iterations to converge, which might be a challenge for large matrices.

**Table 4:** Deviation and correlation between the upper triangle (excluding diagonal) elements of *C*_(5 × 5)_ (the correlation matrix) and its weighted (using *W*_(5 × 5)_) bent matrix

Statistics	HJ03–2^*a*^	HJ03–4^*b*^	LRS14^*c*^
Min (dev.)	−0.2056	−0.1995	−0.1994
Max (dev.)	0.0644	0.0630	0.0632
Mean (dev.)	−0.0293	−0.0284	−0.0284
AAD	0.0569	0.0554	0.0555
Weighted AAD	0.0146	0.0142	0.0142
Correlation	0.9428	0.9463	0.9462
Weighted correlation	0.9939	0.9943	0.9942
RMSD	0.0828	0.0803	0.0804
Weighted RMSD	0.0110	0.0107	0.0108
Number of iterations	88	286	694

dev. = bend(**V**) – **V**; AAD = average absolute deviation; RMSD = root of mean squared deviation; ^*a*^ Method of Jorjani et al. [4] with ϵ = 10^−2^; ^*b*^ Method of Jorjani et al. [4] with ϵ = 10^−4^; ^*c*^ Method of Schaeffer [3]

Like comparing Tables 1, 2 and S1, comparing Tables 3, 4 and S1 shows lower weighted AAD and weighted RMSD, and higher weighted correlation for weighted compared to unweighted bending, at the cost of greater deviations, AAD and RMSD, and lower correlation coefficients.

### The ill-conditioned genomic relationship matrix (G)

**Fig. 1:**
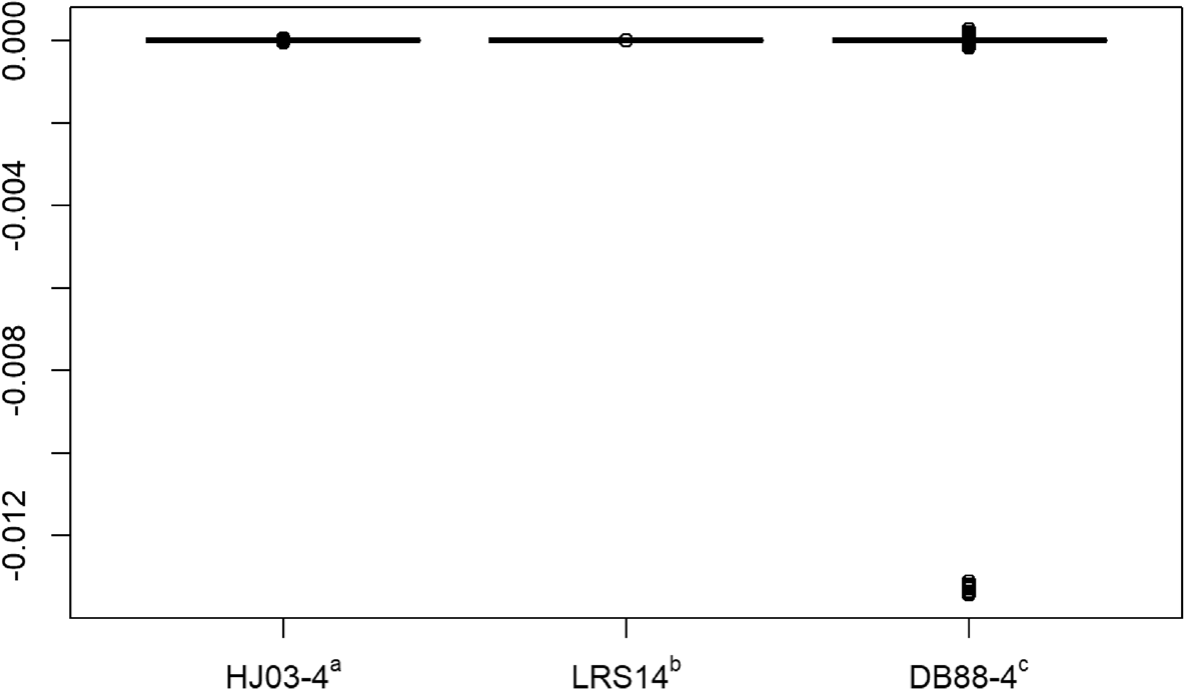
Boxplot of the upper triangle elements of $\hat{G}-G$ for different methods. $\hat{\mathbf{G}}$ = bent **G**; ^a^ Method of Jorjani et al. [4] with ϵ = 10^−4^; ^b^ Method of Schaeffer [3]; ^c^ Method of Bock et al. [7] with ϵ = 10^−4^

The **G** matrix was bent using HJ03–4, LRS14 and DB88–4. Assuming all genotypes of the same quality, unweighted bending was carried out. The distributions of the upper triangle elements of $\hat{\mathbf{G}}-\mathbf{G}$ are shown in Fig. 1, where $\hat{\mathbf{G}}$ is the bent **G**. All the three methods performed well providing minimal deviations. DB88–4 showed larger deviations with 10 elements showing deviations between −0.0131 and −0.0135. LRS14 performed the best. The AAD values were 8.5e–15, 1e–7 and 1.47e–5, and the RMSD values were 11e–15, 4e–7 and 6.17e–5 for LRS14, HJ03–4 and DB88–4, respectively. It took HJ03–4, LRS14 and DB88–4, 6 s, 47 s and 3 s time to derive $\hat{\mathbf{G}}$, in 1, 8 and 1 iterations, respectively.

## Discussion

The covariance (**V**), the correlation (**C**) and the genomic relationship (**G**) matrices were successfully bent using different methods. In situations where the precision of different matrix elements is known (e.g., the number of observations involved or the standard errors), weighted bending is highly recommended. It may cost an overall larger deviation between the original and the bent matrices, but minimising the deviations or preserving the elements with higher precision. The extension of LRS14 to a weighted bending worked perfectly and proved to be better than LRS14. An improvement made to HJ03 was changing **W** to **W**/max(**W**), internally [8]. This reduced the number of iterations to convergence, by changing max(**W**) to 1. This practice is recommended as elements of [**11**′ – **W**] and **W** are positive and convex combinations of each other (i.e., **V**_*n* + 1_ = **V**_*n*_ − [**V**_*n*_ − **U**_*n*_**Δ**_*n*_**U**_*n*_′] ∘ **W** = **V**_*n*_ ∘ [**11** ′  − **W**] + [**U**_*n*_**Δ**_*n*_**U**_*n*_′] ∘ **W**).

The unweighted bending of the covariance matrices (**V** and **G**) took an iteration to converge, except LRS14 for **G**, which took 8 iterations to converge. For the unweighted bending of the correlation matrix, except DB88–4, the other methods converged in more than 1 iteration. The reason was that unweighted bending for correlation matrices is equivalent to a weighted bending (except for DB88–4) with off-diagonal weights equal to 1, and diagonal weights equal to 0. As a result, correlation coefficients between the original and the bent matrix decreased from Table 1 to Table 3 (covariance to correlation), but it increased for DB88–4 from 0.9833 to 0.9896, because this method is mainly designed for bending correlation matrices. Probably for the same reason, it did not perform as good as HJ03–4 and LRS14 for bending **G** (Fig. 1). As a result of DB88 being designed for correlation matrices, when it comes to covariance matrices, the diagonal elements are not allowed to change, which puts more force on the off-diagonal elements to change. Contrary to weighted bending of the covariance matrix, weighted bending of the correlation matrix converged in fewer number of iterations.

The most important statistics to judge among different bending methods are absolute distance measures such as AAD and RMSD. Correlation coefficients between the original and the bent matrix are not important as such but showing the direction of changes corresponding to the value of elements in the original matrix. Where weighted bending is involved, weighted AAD and weighted RMSD should be considered.

Although, LRS14 is not based on any known statistical properties [3], it performed close to HJ03–2 (slightly better for **C** and slightly worse for **V**) and it was the best performer for **G**. In the first iteration of bending **C** by LRS14, the eigenvalues −0.0312 and −0.1852 were replaced with 0.0019 and 0.0007, respectively. Therefore, the benefit over HJ03–2 may come from a combination of these values being in a descending (rather than equal) order and less than 10^−2^. In the first iteration of bending **V** by LRS14, the eigenvalues −3.1229 and −18.5235 were replaced with 0.2036 and 0.0774. Given these values are greater than 10^−2^ (compared to HJ03–2), the benefit in replacing negative eigenvalues with small positive values in a decreasing order becomes evident. It would be interesting to see how LRS14 performs with increasing the denominator (100 *s*^2^ + 1).

### Other methods

There are many other bending methods used in other fields, such as psychology, economics, finance and engineering. Most of those are designed for bending correlation matrices. Therefore, applying them to covariance matrices would result in unchanged diagonal elements and consequently further changes in off-diagonal elements. Several of those methods are explained by Marée [11], and Lorenzo-Seva and Ferrando [13]. As examples, here, a few methods are explained briefly.

Rebonato and Jäckel [14] used hypersphere decomposition methodology for creating a valid correlation matrix for the use in risk management and option pricing. In this trigonometric-based method, $\hat{\mathbf{C}}=\mathbf{BB}^{\prime }$ and the row vectors of **B** are coordinates of angles (*θ*_*ij*_) lying on a unit hypersphere. The elements of **B** are calculated as:

$$ {b}_{ij}=\left\{\begin{array}{ll}\cos {\theta}_{ij}& \mathrm{for}\ j=1\\ {}\cos {\theta}_{ij}.{\varPi}_{k=1}^{j-1}\sin {\theta}_{ik}& \mathrm{for}\ j=2\ \mathrm{to}\ n-1\\ {}{\varPi}_{k=1}^{j-1}\sin {\theta}_{ik}& \mathrm{for}\ j=n\end{array}\right. $$

Rapisarda et al. [15] simplified this method by reducing **B** to a lower triangle matrix. This method resembles deriving the Cholesky decomposition of a PD correlation matrix close to the non-PD correlation matrix. The elements of **B** are calculated as:

$$ {b}_{ij}=\left\{\begin{array}{ll}1& \mathrm{for}\ i=j=1\\ {}\cos {\theta}_{ij}& \mathrm{for}\ i\ge 2,j=1\\ {}{\varPi}_{k=1}^{j-1}\sin {\theta}_{ik}& \mathrm{for}\ i=j,2\le i\le n\\ {}\cos {\theta}_{ij}{\varPi}_{k=1}^{j-1}\sin {\theta}_{ik}& \mathrm{for}\ 2\le j\le i-1\\ {}0& \mathrm{for}\ i+1\le j\le n\end{array}\right. $$

Numpacharoen and Atsawarungruangkit [16] introduced a method for obtaining the theoretical bounds of correlation coefficients and an algorithm for permuting random correlation matrices within those boundaries.

Bentler and Yuan [17] developed a bending method via off-diagonal scaling of the matrix. The symmetric PD matrix is obtained as $\hat{\mathbf{V}}=\boldsymbol{\Delta} \left(\mathbf{V}-{\mathbf{D}}_V\right)\boldsymbol{\Delta} +{\mathbf{D}}_V$, where **D**_*V*_ =  *diag* (*diag*(**V**)), **Δ** is a diagonal matrix such that **0** < **Δ**^2^ *diag* (*diag*(**V** − **D**)) < **D**_*V*_, and **D** is a diagonal matrix such that **V** – **D** is PD. **D** can be a diagonal matrix of small negative values, or according to Bentler and Yuan [17], it can be obtained by minimum trace factor analysis [18, 19].

An alternative approach to bending is fitting a reduced rank factor-analytic model. Multi-trait BLUP can be reformulated by changing the covariance structure among *n* traits to the factor-analytic structure of *n* orthogonal factors [20]. In a reduced rank factor-analytic model, specific factors (not explaining the common variance) are absent, by setting the corresponding eigenvalues to zero [20].

Obviously, there should be some confidence around matrix elements. Neither a rank reduced factor-analytic model nor bending can solve the problem of major flaws in matrix elements. For both methodologies, the reference is the original matrix. In bending, only if uncertainty around some matrix elements causes non-PDness, the matrix is bent with as minimal as possible impact on the original matrix. Similarly, when it comes to **G** matrix, prevention (i.e., quality control and discarding problematic genotypes) is better than cure (i.e., bending).

## Conclusions

This study introduced a new R package for bending symmetric non-PD matrices to PD, with the flexibility of choosing between weighted and unweighted bending, two different bending methods, the smallest positive eigenvalue (for one of the methods), and direct or reciprocal use of the weight matrix elements for weighted bending. Together with the bent matrix, several bending performance statistics are provided by the program. Where precision of matrix elements is available, weighted bending is recommended. There was benefit in small positive values in a descending order replacing negative eigenvalues. This method can further benefit from the possibility of choosing the smallest positive eigenvalue, which can be a topic for future research. The differences between the performance of different methods were minor and the methods ranked differently for different matrices. Therefore, testing different methods, and ϵ values (for HJ03) are recommended. Bending methods may perform differently for different matrices, depending on whether a covariance or a correlation matrix is being bent, the number of negative eigenvalues and their magnitude relative to the smallest positive eigenvalue, and the size of the matrix (i.e., number of diagonal elements relative to all elements).

There are many bending methods available, and those have approached the problem in various ways. Some methods are preferable for correlation matrices. This study showed that eigendecomposition-based methods are simple, robust and computationally efficient. Finally, the application of bending and R package mbend is not limited to multi-trait BLUP, but also other multivariate mixed models, genetic selection indices [5], or any situation, where a symmetric non-PD matrix needs to be transformed to a PD matrix.

## Availability and requirements

- **Project name:** R package mbend
- **Project home page:** https://cran.r-project.org/package=mbend
- **Operating system(s):** Platform independent
- **Programming language:** R
- **Other requirements:** None
- **License:** GPL-3
- **Any restrictions to use by non-academics:** None

## Supplementary information

**Additional file 1 Appendix**. Bending a correlation matrix using function bend from R package mbend. **Table S1**. Weighted statistics (using *W*_(5 × 5)_) between the upper triangle elements of *V*_(5 × 5)_ (the covariance matrix) and *C*_(5 × 5)_ (the correlation matrix) and their unweighted bent matrices.

## Data Availability

The datasets and code supporting the conclusions of this article are available in the Mendeley repository: 10.17632/kf3d8v8939.1.

## Abbreviations

BLUP: Best linear unbiased prediction
HJ03: Method of Jorjani et al. (2003)
HJ03–2: HJ03 with ϵ = 10^−2^
HJ03–4: HJ03 with ϵ = 10^−4^
LRS14: Method of Schaeffer (2014)
DB88: Method of Bock et al. (1988)
DB88–4: DB88 with ϵ = 10^−4^
