# Supplementary material for: mbend: an R package for bending non-positive-definite symmetric matrices to positive-definite
Source: BMC Genet. 2020 Sep 3;21:97. doi: 10.1186/s12863-020-00881-z (PMC7469428; doi:10.1186/s12863-020-00881-z)
Supplement: Supplementary file 1 — Additional file 1 Appendix. Bending a correlation matrix using function bend from R package mbend. Table S1. Weighted statistics (using W(5 × 5)) between the upper triangle elements of V(5 × 5) (the covariance matrix) and C(5 × 5) (the correlation matrix) and their unweighted bent matrices. [file 12863_2020_881_MOESM1_ESM.pdf]

Appendix

Bending a correlation matrix using function `bend` from R package mbend.

```
> V = matrix(nrow = 5, ncol = 5, c(
+   100,  95,  80,  40,  40,
+    95, 100,  95,  80,  40,
+    80,  95, 100,  95,  80,
+    40,  80,  95, 100,  95,
+    40,  40,  80,  95, 100))
> C = V/100
> W = 1/matrix(nrow = 5, ncol = 5, c(
+   1000, 500,  20,  50, 200,
+    500,1000, 500,   5,  50,
+     20, 500,1000,  20,  20,
+     50,   5,  20,1000, 200,
+    200,  50,  20, 200,1000))
> bend(C, W)
Weighted bending
reciprocal = FALSE
max.iter = 10000
small.positive = 1e-04
method = hj
Found a correlation matrix.
Convergence met after 286 iterations.
$bent
          [,1]      [,2]      [,3]      [,4]      [,5]
[1,] 1.0000000 0.9447536 0.8342673 0.4377103 0.3912583
[2,] 0.9447536 1.0000000 0.9385087 0.6004642 0.4630373
[3,] 0.8342673 0.9385087 1.0000000 0.8394178 0.7248761
[4,] 0.4377103 0.6004642 0.8394178 1.0000000 0.9418815
[5,] 0.3912583 0.4630373 0.7248761 0.9418815 1.0000000
$init.ev
[1]  3.99475997  0.98523500  0.23646897 -0.03122893 -0.18523500
$final.ev
[1] 3.868843e+00 1.015420e+00 1.156566e-01 7.740771e-05 2.031735e-06
$min.dev
[1] -0.1995358
$max.dev
[1] 0.06303735
$loc.min.dev
row col
  4   2
$loc.max.dev
```

```
row col
  2   5
$ave.dev
[1] -0.02838248
$AAD
[1] 0.05538548
$Cor
[1] 0.9463128
$RMSD
[1] 0.0803483
$w_gt_0
[1] 10
$wAAD
[1] 0.01416959
$wCor
[1] 0.9942541
$wRMSD
[1] 0.01074558
```

Table S1: Weighted statistics (using $\mathbf{W}_{(5\times5)}$) between the upper triangle elements of $\mathbf{V}_{(5\times5)}$ (the covariance matrix) and $\mathbf{C}_{(5\times5)}$ (the correlation matrix) and their unweighted bent matrices

Matrix	Statistics	HJ03-2[a]	HJ03-4[b]	LRS14[c]
$\mathbf{V}$	Weighted AAD	3.9753	3.9718	4.0202
	Weighted corelation	0.9736	0.9736	0.9734
	Weighted RMSD	4.5509	4.5474	4.5901
$\mathbf{C}$[d]	Weighted AAD	0.0479	0.0465	0.0481
	Weighted corelation	0.9913	0.9920	0.9923
	Weighted RMSD	0.0483	0.0469	0.0482

AAD = average absolute deviation; RMSD = root of mean squared deviation; [a] Method of Jorjani et al. (2003) with $\epsilon = 10^{-2}$; [b] Method of Jorjani et al. (2003) with $\epsilon = 10^{-4}$; [c] Method of Schaeffer (2014); [d] Excluding diagonal elements
